# Cancer and its Treatment in Main Ancient Books of Islamic Iranian Traditional Medicine (7th to 14th Century AD)

**DOI:** 10.5812/ircmj.4954

**Published:** 2012-12-06

**Authors:** Seyed Ahmad Emami, Amirhossein Sahebkar, Nilufar Tayarani-Najaran, Zahra Tayarani-Najaran

**Affiliations:** 1 Department of Pharmacognosy, School of Pharmacy, Mashhad, University of Medical Sciences, Mashhad, IR Iran; 2 Biotechnology Research Center and School of Pharmacy, Mashhad University of Medical Sciences, Mashhad, IR Iran; 3 Department of Dental Prosthesis, School of Dentistry, Mashhad, University of Medical Sciences, Mashhad, IR Iran; 4 Department of Pharmacology and Pharmacological Research Centre of Medicinal Plants, School of Medicine, Mashhad University of Medical Sciences, Mashhad, IR Iran

**Keywords:** Cancer, Traditional, Medicine, Islamic Medicine

## Abstract

**Abstract:**

Islamic medicine is regarded as a comprehensive medical school with a long, glorious and worldwide reputation. Some of the physicians of this school are famous worldwide and have contributed valuable services to the scientific world. Given the dramatically increasing prevalence of cancer and the relative inefficacy of current medications, there is a great demand for the introduction of effective therapeutic approaches. To this end, integration of traditional medicine with modern medical treatments represents a promising option. In this essay, methods of diagnosis and treatment of cancer have been mentioned from the viewpoint of five famous physicians before the Mongolian attack who used Islamic medicine, namely Rhazes, Akhaveyni, Ahwazi, Avicenna and Jorjani. The ideas discussed dates back to a period between the eighth and fourteenth centuries.

## 1. Introduction

Islamic medicine is a holistic and comprehensive medical school that has an antecedent over 12 centuries. Using the scientific knowledge of ancient Iran, ancient Greece, and archaic civilizations such as India and China, supplemented useful and wise Islamic thaughts, Islamic medicine has turned into a strong and permanent medical school. Islamic medicine has, for many centuries, been used for diagnosing and treating diseases of large populations that live in vast geographic areas. Some of the physicians of this school are famous worldwide and have contributed valuable services to the scientific world.

In this writing, we will discuss cancer and the ways to its diagnose and treatments from the viewpoint of a few of the most famous physicians before the Mongolian attack who practiced Islamic medicine. The ideas discussed here dates back to the era between the eighth and fourteenth centuries. The interesting point is that all of the physicians mentioned in this writing are Iranian:

Abu Bakr Mohammad ibn Zakariya Razi, known as Rhazes (251-313 A.H./865 – 925 A.D.), the renowned Iranian physician, philosopher and chemist who wrote about 250 books and treatises;

Abu Bakr Rabi ibn Ahmad Akhaveyni Bukhari who is one of the renowned physicians and the student of Abu al-Qassem Moqanei (a Rhazes’ student). He died in 373 A.H. (983 AD);

Ali ibn Abbas Majussi Ahwazi Arrajani , the most noted Muslim physician after Rhazes was known as Haly Abbas to the westerners (338-384 A.H./948 - 994 A.D. );

Shaykh al-Ra’is (Supreme Guide) Abu Ali Hussain ibn Abdullah ibn Sina known as Avicenna (370-427 A.H./980-1037A.D.), who is the most prestigious scholar of Iran and the world of Islam. He emerged after Ahwazi;

Seyyed Esma’il Jorjani (434-531 A.H./ 1042-1136 A.D.) who is regarded as the most important celebrated physician after Avicenna.

**Figure 1:**
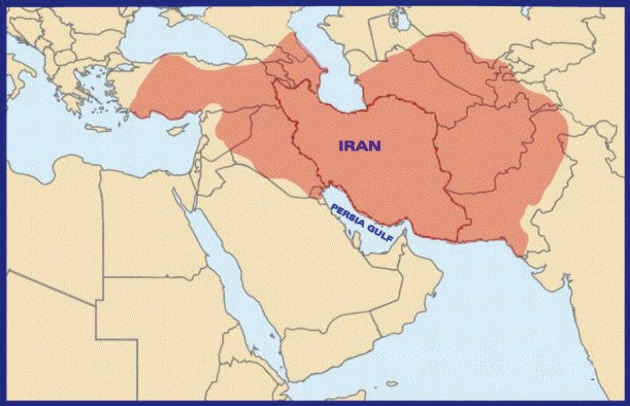
Territory of Ancient Iran in the Sixth Century A.H.

Due to the vast territory of the ancient Iran (Figure 1), these physicians are regarded as the main icons of historical medicine in many countries of the Middle East region. Almost all reviewed books (except the one compiled by Zakhireh Kharazmshahi) are written in Arabic and have been translated into several other languages including Persian, Turkish and Hebrew.

## 2. Results and Discussion

### 2.1. Rhazes

Al-Hawi (The Continens) is Rhazes’ most important and most inclusive book. Rhazes spent 15 years on this book. The book was translated into Latin in 1279 by Faraj ibn Salem (Farrgut) and was reprinted five times in Europe between 1488 and 1542. The Arabic text of Al-Hawi was published in Heydarabad, India, in the 7th decade of the 20th century. Among other famous medicinal books of Rhazes the following can be mentioned:

1) Man la Yahduruhu al-Tabib (for One without Doctor), a medical advisor for the general public. Rhazes was probably the first Persian doctor to deliberately write a home medical manual (remedial) directed at the general public. The contents of this book are covered through 36 chapters. 

2) Al-Mansouri: written in 10 chapters. In al-Mansouri, Rhazes has presented a description of the identification of tempers, anatomy, hygiene, orthopedics, wounds and sores, bites and a complete course of therapeutics. This book was translated into several European languages and was published many times.

3) Al-Jodari wa al-Hasbah (Smallpox and Measles) which was the first book on differential diagnosis of smallpox and measles. It was reprinted more than forty times in Europe. 

4) Al-Morshed (The Guide) which includes 29 chapters and is an adaptation of one of Hippocrate’s writings. 

Some other of his medical books are al-Tibb al-Mlouki (Royal Medicine), Bur al-Sa’ah (Medical Emergencies), al-Taqseem wa al-Tashjir (Divisions and the Branches), al-Qarabadin al-Kabir (The Great Book of Dispensatories) and al-Shukuk al’a Jalinus (Doubts about Galen). Rhazes was the most important specialist in clinical and practical medicine in the Islamic world (1-6).

In the first section of this writing, Rhazes’s view about cancer is described. In his famous book, Alhawi, he has described the views of the scientists who lived before him and in between, together with his own opinions (7):

Galen has quoted from Dioscorides that applying a poultice prepared from hedge mustard (Erysimum officinale L.) is useful for the treatment of non-ulcerative cancer. Paul of Aegina has noted that applying the aforementioned poultice is effective against parotid cancers. Galen has claimed that hedge mustard causes inflammation, has a taste similar to garden cress (Lepidium sativum L.) and is beneficial in the treatment of otitis as well as indurated swellings of breasts and testicles. According to Galen, nettle (Urtica dioica L.) has efficacy in the treatment of corrosive cancers, a feature which can be attributed to the non-stinging astringent effects of this herb. Dioscorides has mentioned that applying the inner crust of walnut (Juglans regia L.) on ulcerative melatonic swellings is a useful therapeutic approach.

Rhazes' experience: Rubbing the lotion prepared from basic carbonate of lead is effective, chicory (Cichorium intybus L.) juice and a small amount of opium against ulcerative, pulsating and warm cancer with many rashes, and helps relieve its warmth and pulsation. 

Rhazes' experience: Eating the cooked mixture of viper's meat, water, salt, dill (Anethum gravolens L.) and wine made from fragrant herbs is effective in the treatment of newly developed cancer. Viper's meat has the same effect as well. In addition, poultice of water cooked pea (Cicer arientium L.) promotes healing of cancerous wounds. Galen and Dioscorides have mentioned that milk, either alone or in combination with analgesic drugs, could relieve the pain associated with different kinds of cancerous wounds. The best drug that could be mixed with milk for this purpose is washed zinc oxide. Galen and Dioscorides have also quoted that loferghesh has the same property and its analgesic effect is superior to that of mineral drugs. Galen has mentioned that the effect of dressings prepared from powdered lead and cold extracts is very beneficial against ulcerative cancers. Another finding of Galen is that sprinkling burnt lead, particularly in the washed form, is beneficial for the recovery of ulcerative cancers.

According to Galen, sprinkling the sifted powdered old woods of goat willow (Salix caprea L.) on cancerous wounds in the morning and at night is a very effective approach. Besides, washing these wounds with the decoction of oriental plane tree (Platanus orientalis L.) leaves is very beneficial. After washing with the aforementioned decoction, cancerous wounds should be covered with dwarf mallow (Malva rotundifolia L.) leaves. 

Slemon has said that black bile purgatives are effective in the treatment of cancer and everything that moistens the body should be implicated in the suggested nutritional diet of cancer tissue. He has also pointed that administration of antidote, in particular Electuarium Mithridatium, is efficacious in cancer therapy. Consumption of donkey (Equus africanus asinus L.) milk and rubbing with non-hot-tempered emollient balm have also been suggested to prove beneficial.

In his book entitled "Methods of Treatment", Galen has hypothesized cancer as a disease associated with black bile humor which is very hard to be diagnosed at early stages. In order to treat cancer, Galen has proposed that black bile should be removed from the body by means of administering an appropriate purgative, and then preventing the generation and accumulation of black bile in vessels as far as possible. If this method is not applicable, black bile should be removed from the body at regular time points. A mixture of 17.84 g clover dodder (Cuscuta epithymum Murr.) with cheese whey should be used in order to boost organ's function and black bile removal. Topical anti-cancer drugs should have moderate lytic activity as drugs with mild activity cannot dissolve the phlegm and those with strong activity will dissolve the soft parts of the phlegm and make the remaining parts tough and hard. Aside from moderate lytic activity, drugs should not be caustic because cancer is a malignant disease and should not be treated with irritant drugs. Therefore, administration of caustic and irritant agents will stimulate the disease.

Administration of the aforementioned drugs together with some black bile purgatives would lead to recovery at the early stages of disease. However, in case of advanced cancers, disease progression should be prevented by all means at hand. If surgery is to be performed, removing black bile should be regarded as the first priority and must be done as completely as possible. Then, tumor should be removed in a way that no root is left behind. Bleeding should be allowed with no haste in stanching. Afterwards, adjacent vessels should be pressed in order to remove their thick blood. The formed wound should be treated afterwards. Galen has also noted that the cancerous organ or other malignant non-healing ulcers should be cut.

In one of his books, Galen mentions that cancer development is due to the black bile blood. He notes the rationale for this hypothesis as follows: First, the blood in cancer tissue is black. Second, cancerous organ is not warm in physical examination. Third, vessels in the cancerous tissues are darker and have more blood content compared to tissues with warm swelling. Galen continues that cancerous tissues are more malignant if accompanied by wounds, otherwise they tend to be benign.

According to Jew (Masarjawai), cancer is frequently formed in the uterus, breast, and eyes. Galen has mentioned in the book “Purgative Drugs” that there is a possibility of treating cancer and malignant wounds by means of only administering purgatives. Sergius of Reshaina has noted that when thin blood flows from the uterus for a long period, there is the possibility of cancer formation in the mentioned organ. The reason is that in such cases the thick portion of blood will remain in the uterus and cause cancer. Likewise, flow of thin milk from breast for a long period indicates the possibility of breast cancer.

In the book entitled “Thick Substances with Abnormally High Concentrations”, Galen has noted that cancer is associated with black bile humor and when the aforementioned humor is warm, it will lead to ulcerative cancer. He has also added that cancerous tissues have darker appearance and lower temperature compared to warm swellings. Besides, the vasculature of cancerous tissue is hyperemic and contains higher and darker blood content compared to other types of swelling. In case of small ulcerative cancers in non-vital organs, venesection should be performed following repeated administration of purgative drugs. Afterwards, caustic drugs should be placed to eradicate cancer. One should not that this method should not be performed for other types of cancer.

It has been mentioned in the “al-Fosool” book that it is better to leave latent and asymptomatic cancers untreated in order to prolong patient’s life. Intervention in these cases may increase the risk precocious death. Latent cancer in this context refers to non-ulcerative cancers and cancers of internal organs and viscera.

Galen has noted that some types of cancers could be recovered through surgery and cauterization. Rhazes mentions that: “as far as I am aware, internal cancers are not recoverable and treatment of these cancers would accelerate patient’s death. I have observed cases with palate, anal or vaginal cancers in which surgery and wound cauterization prevented wound healing and caused patient’s torment till death. Apparently, if these patients were left untreated, they would have a longer life and would not have undergone treatment related torments.” Thus, the aforementioned types of cancer should not be treated unless they are ulcerative and have secretion. For the treatment of superficial cancers, all cancer roots, i.e. adjacent vessels that are full of dark blood, should be cut. However, many physicians have argued that these approaches should only be used for cases in which cancer has irritating wounds and/or involved an organ that is possible to be cut and cauterized, as well as cases in which the patient is determined and willing for cutting vessels. Sprinkling walnut gum on ulcerative cancer is very beneficial. Abujarih has also approved the efficacy of this remedy.

According to Athenaeus of Attalia, rubbing the mixture of whitened ash - obtained from burning an aquatic turtle - and ghee on ulcerative cancer would cleanse the wounds, accelerate their healing process and prevent their relapse. The aforementioned drug is effective against all types of wounds as well as heat burns. Athenaeus has also pointed that rubbing the rennet obtained from rabbit (Lepus capensis L.) has wonderful effects on ulcerative cancer. In addition, he believed that applying the mixture of antler ash and human milk as an ointment on newly formed cancer is efficacious.

In the book “al-Ayn” (the eye), Galen has mentioned that if cancer is diagnosed at its early stages, though not very much common, its treatment would be possible. As cancer progresses, there would be no options but cutting the affected tissue. However, surgery and organ excision may raise serious problems including severe bleeding seen particularly in large tissues with high vessel density, severe pains in vital organs which is due to the high amount of moisture removed from dissected vessels, and impossibility of surgery for structures adjacent to vital organs. In contrast, cancer could be treated in its early developmental stages by administering purgatives such as clover dodder and cheese whey. Patients who suffer from these types of cancers should consume wet, soft and cool foods capable of attenuating black bile-induced burning. Some examples of these foods that could help cancer treatment or halt its progression are squarters goosefoot (Chenopodium album L.), pumpkin (Cucurbita pepo L.) and little fishes.

Antyllus has described cancer as a kind of spherical swelling with deep and hyperemic adjacent vessels that could be considered as cancer feet. He has also mentioned that metastatic cancer has stringent and lethal pain, sensible warmth upon prolonged physical examination of the tumor and swollen and inflamed adjacent vessels. Antyllus points out that cancerous wounds have inward corrosiveness, liquid and stinky pus and two thick and erythematous edges. If these wounds are deep or placed in an organ which is not possible to be cut, neither should they be treated nor manipulated and only palliative relief should be considered. If the cancer is in one of nostrils, fingers or their adjacent areas, or breast, tumor should be eradicated (if possible) and after considerable bleeding the wound should be cauterized afterwards.

In his book titled “Methods of Treatment”, Galen has mentioned that cancer is hard to detect at its early stages. He has also added that newly formed cancers are curable through removal of harmful phlegms and applying some topical drugs. In the case of advanced cancer, the only plausible measure is to prevent the progression. If the physician dares to operate such type of cancer, harmful phlegms must firstly be removed from the body. Afterwards, eradication of cancerous tumor should be attempted in a way that all tumor roots are cut. Then, adjacent vessels should be pressed in order to remove their thick blood.

Rhazes’ experience implies that some hard swellings are similar to cancer. These swellings are categorized into those with and without sense. Differentiation of such swellings is based on the fact that hard swelling is usually secondary to warm swelling (such as phlegmatic or similar swellings), is dependent to other phenomena and is never formed initially. In contrast, cancer is formed primarily. Another issue is that the vessels adjacent to non-cancerous swellings are stretched and have lower temperature upon touch compared to cancerous tumors. For senseless swellings, this is the best sign of their non-cancerous nature.

In the "Semeiolgy" book it has been mentioned that cancer is primarily a small and mobile swelling similar in shape to broad bean. It could sometimes be enlarged to the size of a walnut and larger, thereby losing its mobility. Such large tumors are very sensitive and painful, with a distinctive red to yellow color and their pain is caustic and burning. Such tumors might rupture spontaneously revealing their infectious and blood-like content. The resulting wounds are very sensitive and could digest and invade neighboring tissues. If potent drugs are applied on the aforementioned wounds, convulsion, fever, fainting and chills will occur and secreted pus will irritate adjacent tissues.

According to the book “Summary of Treatment Methods”, black chyme should be removed from the body as far as possible in order to prevent growth and progression of early cancers. Black chyme is formed during the early stages of cancer development.

Rhazes’ experience: during the initial phases of cancer, regular venesection and administration of black bile purgatives is suggested. In addition blood thinning foods with cold nature should be administered for the patient.

Masarjawai has mentioned that in the case of ulcerative cancers, the balm prepared from starch, zinc oxide, frankincense (Boswellia carteri Birdew.), aloe (Aloe spp.), red Armenian bole and rose oil should be dressed on the cancerous wound.

According to Aaron of Alexandria, the balm prepared from pulverized starch, sponge (Spongia officinalis L.), basic carbonate of lead, black nightshade (Solanum nigrum L.) water and rose oil should be applied for the treatment of cancerous wounds.

Paul has noted that the prevalence of cancer is higher among females which are due to their generally weaker stamina and lower tolerance to concentrated wastes. He also adds that cancer is more prevalent in some organs such as the neck, breast and nervous organs. The cancerous area should be dressed with a piece of damp cloth soaked in black nightshade extract and when the cloth becomes dried, it should be resoaked with the same extract. Poultices prepared from lettuce (Latuca sativa L.) extract, common houseleek (Sempervivum tectorum L.) and powdered zinc oxide are also effective. Besides, powdered red Armenian bole could be mixed with any of the aforementioned extracts and the resulting poultice could be applied on the cancerous area. Cancer patients should not consume thick foods. Instead, they should use cold and moisturizing foods such as cucumber, beer, cheese whey, sumac (Rhus coriaria L.), purslane (Portulaca oleracea L.) and young fish and bird meat. Oribasius has suggested the following remedy to be very effective against corrosive cancers:

Sumac and cassia [Cinnamomum cassia (L.) J. Presl] should be soaked in astringent wine for 4 days, then boiled and mixed with Mediterranean cypress (Cupressus sempervirens L.) wood. The mixture is then condensed and filtered followed by reboiling. When the mixture finds a honey-like viscosity, heating is stopped and the mixture should be kept in glass containers. Rubbing the above balm on corrosive wounds has an excellent effect on their healing. In addition, application of this balm is also very efficacious against progressive wounds. 

According to Paul, cancer is a kind of sensitive and painful swelling which has black color, ugly and irregular appearance which could be ulcerative. Furthermore, cancerous tissue has vessels stretched in different directions. When formed in an organ which could be cut, cancer should be eradicated and its scar be cauterized.

According to Aristoxenus, cancer is a kind of spherical swelling that, upon initiation of treatment, will start to progress. Warmth is a characteristic of cancer which could be sensed upon prolonged touch. Hyperemic vessels exist near the cancerous swelling. The main mass and the inflamed areas are located in depth. In ulcerative cancers, thin, stinky and corrosive pus is secreted. The wound resulting from tumor rupture has hard and red edges and the physicians do not frequently dare to cut it, unless it is situated in organs such as the nose and fingers. If cutting the tumor is applicable, it should be removed deeply and with some portions of normal adjacent tissues and then be cauterized in order to prevent its recurrence. Afterwards, some balms could be applied on the scar to eliminate the formed slough.

Rhazes’ experience: In case of cancer, black bile purgatives should be administered 10 times per week and appropriate body moisturizing measures is applied. If there is a large vessel near the cancerous area, it should be venesected. Afterwards, dissolvent drugs, cooling agents and damp cloth should be placed on the tumor. In case of wound formation, mild corrosive drugs such as yellow vitriol (ferric oxide) and verdigris (basic acetate of copper) should be sprinkled with caution not to intensify the wound’s pain.

Galen and Dioscorides have mentioned that hoary stock [Matthiola incana (L.) W.T.Aiton] poultice is effective against non-ulcerative cancers. Galen has noted that the aforementioned poultice is greatly effective in the elimination of hard swellings especially those in the breast and testicles. Moreover, dragon wort (Arum dracunculus L.) seeds have a highly dry nature and are therefore effective in the treatment of chronic cancers. Galen has added that hedge mustard – a plant with leaves similar to those of rocket (Eruca sativa Mill.), narrow branches, yellow flowers and fine seeds – is effective against non-ulcerative cancers and all types of hard swellings. It has also been mentioned that the poultice prepared from Gundelia (Gundelia tournefortii L.) gum and flax (Linum usitatissimum L.) mucilage would eliminate cancerous swellings.

Physicians from Khuzestan have mentioned that pea flour poultice is effective in the treatment of cancer. They have also noted that the poultice prepared from fat and burned cabbage (Brassica oleracea L.) roots could gradually eliminate cancer. In case of tumor irritation, chicken fat should be applied for some days until irritation is relieved. Afterwards, the treatment should be repeated. Venesection, administration of purgatives, consumption of moisturizing foods and bathing are also suggested. It has been mentioned that common plantain (Plantago major L.) poultice is effective against many types of cancers.

Rhazes’ experience: Whenever there is a doubt about the cancerous nature of a tumor, it should be touched by hand for a long period of time and if warmth is sensed, the tumor is most likely cancerous.

Rhazes’ experience: The temperature of scrofulous tumor is lower or equal to that of the body.

Qusta ibn luqa has said that apostles’ ointment treats cancer. Archigenes has noted that in the early stages of cancer, poultice prepared from equal amounts of gold or silver litharge (impure oxide) and river crab (Liocarcinus vernalis Risso) could be applied to the affected area. Besides, ash obtained from the river crab could be mixed with wax and oil to form a paste which could be then applied to cancerous area. In case of ulcerative cancers, application of paste from vinegar, sealing clay (Terra sigillata) and powdered lead has been suggested. For these cancers, application of poultice obtained from black nightshade juice is also effective.

In page 73 of the 2nd volume of al-Hawi, Rhazes has mentioned points about eye cancer. He has discussed nose (pages 72-75 of 3rd volume), breast (11-14 of 7th volume) and liver (89-100 of 7th ) cancers in his book as well. In page 11 of the 8th volume and pages 297-314 of the 11th volume of al-Hawi some issues regarding intestinal and uterus cancers have been provided, respectively.

### 2.2. Akhaveyni Bukhari

Akhaveyni dedicated his whole lifetime to medicine. He recorded his medicinal attempts in Hedayat al-Mota’allemin fi al-Tibb (An Educational Guide for Medicinal Students). The book, written in an eloquent Persian language, contains three parts:

The first part includes 51 chapters on elements, tempers, humors, simple and compound organs together with descriptions on functions, souls, foods and drinks, physical movement and rest, sleep etc.

The second part, in 130 chapters, applies pathology cap-a-pie. In the third part, consisting of 19 chapters, he has introduced various types of fevers and pulses. This book was published by Ferdowsi University of Mashhad in Iran in 1965 (2, 3, 5).

Akhaveyni has assigned a chapter of his book “Hedayat al-Mota’allemin” to cancer. He states that cancer is curable during its early stages and its surgery and cutting should be performed whenever possible. However, incomplete cutting or cauterization of a tumor is never suggested as it might cause the patient's death.

When cancerous tumor is formed following warm swellings, treatment or stopping its progression is possible. The tumor is initially in the size of a broad bean. It will then begin to grow gradually and reaches to the size of a walnut or larger and becomes hard and a bit warm. The primary treatment measures include venesection, black bile purgation, consumption of easily digestible foods (such as chicken meat, lamb meat, fresh milk and almond oil) and application of cooling drugs such as ispaghula (Plantago ispaghula Roxb.), tin oxide, basic carbonate of lead, vinegar and red Armenian bole, which prevent the progression and tumor induced harm. In case of tumor injury, marsh-mallow (Althaea officinalis L.), or camphor [Cinnamomum camphora (L.) T.Nees and C.H.Eberm.] balm should be applied. The characteristic pus secreted from ulcerative cancerous tumors is its dark color, stinky smell and black or red openings (8). Akhaveyni has also mentioned the signs and treatment methods of uterine cancer in page 537 of his book.

### 2.3. Ahwazi

Ahwazi is the author of the valuable book Kamil al-Sina’ah al-Tebbiyyah (Complete Book of the Medical Art) or al-Maliki. The al-Maliki is divided into 2 parts. Each part contains 10 discourses which cover the complete course of medicine. The first ten deal with the theory of medicine and its divisions and also types of tempers, elements, humors, anatomy, physiology, general principles of hygiene, diseases and their divisions, types of pulses, kinds of fevers, symptoms of diseases cap-a-pie and subjects on the period and consequences of diseases. The second ten contain topics on health and hygiene care, introductions to all kinds of therapeutic methods, treatment of different types of fevers, dermatologic ailments, all kinds of bites and poisonings, headaches and psychological diseases, respiratory diseases, heart diseases, gastrointestinal diseases, and urogenital diseases, a complete course on surgery and orthopedics, and finally a course on pharmacology and pharmaceutics. The Latin translation was published three times in Europe and the Arabic text was printed in Bulaq, Egypt (1-3, 6).

Ahwazi has described cancer as a kind of swelling that is formed by black bile and in case of progression, has no treatment and is irremediable. Having it eradicated should be considered a priority and must only be chosen for cases without crucial organ involvement. Surgical intervention in cases with vital organ involvement might injure the tumor and transform it to a non-healing wound. Cancerous tumors manipulation and surgical operation is risky and dangerous as they carry the risk of injuring large vessels and arteries in the affected organ with subsequent cancer metastasis to other sensitive and vital organs. Besides, closing such vessels and arteries might cause metastasis to the sensitive organs from which these vessels have originated. Cauterization of the cancerous organ is also a risky measure. If the cancerous swelling is diagnosed at its early developmental stages and other conditions such as age, temper and etc. are favorable, the adjacent vessel should be venesected. If the patient is female, menstruation-inducing drugs should be administered before any other measure. Once the menstrual period is induced, the body should be cleaned up by means of administering black bile purgatives such as dodder and white agaric (Polyporus officinalis Fries) etc. An important note in this regard is that the aforementioned drugs should be administered repeatedly (not just 1-2 times) in order to cleanse the body from black bile. Black bile has a cold and dry nature and is therefore difficult to be moved in the body. One of the effective drugs for cleansing the body from black bile is the following pill:

Black myrobalan (Terminalia chebula Retz.) (3.34 g), dodder (4.01 g), common polypody (Polypodium vulgare L.) (4.01 g), French lavender (Lavandula stoechas L.) (4.01 g), nafti salt (1.14 g) and black hellebore (Helleborus niger L.) (1.67 g) that should all be pulverized, pasted and then formed into a pill. A portion equivalent to 10.02-13.36 g of the pill should be used. After complete cleansing of the body from black bile, appropriate measures (with moderate to wet nature) should be taken to relieve the violence and pungency of black bile until proper blood is produced in the body. In addition, the patient should live in regions with moderate climate and use foods with good chyme such as blite (Amaranthus blitum L.) and pumpkin. Consumption of beer, cheese whey and black bile purgative powders is also suggested.

As for topical medications, the first measure that should be taken before black bile vomit is the application of moderate drugs such as black nightshade, chicory juice, bladder cherry (Physalis alkekengi L.), and similar drugs on the cancerous organ. After cleansing the body from black bile, especially if cheese whey or dodder were used, drugs with moderate lytic activity should be applied. One such a drug is zinc oxide which has the following formula:

Equal amounts of powdered and washed Kermanian zinc oxide, litharge of lead and lead basic carbonate are mixed, gently pulverized and filtered through a silk cloth. The oily part of the balm is prepared by melting wax in rose oil (1:4 ratios). Then, the powder and oil phase are mixed to obtain the balm.

Yellow vitriol balm, cinnabar (mercuric sulphide) balm and apostles’ ointment are other topical medications that could be used for the treatment of cancer and other indurated swellings. Drugs with mild lytic activity are not effective against black bile as this bile is very thick. On the other hand, drugs with high lytic activity would dissolve weak phlegms. Therefore, thick phlegms would accumulate and harden, forming stones that could not be easily dissolved.

In case of tumor injury, application of the following balm is suggested: equal portions of basic carbonate of lead and washed zinc oxide should be mixed with a mixture of rose oil and black nightshade juice [blite juice or coriander (Coriandrum sativum L.) juice could be used, alternatively]. The resulting balm should be applied on the cancerous tumor. Application of the balm mentioned above on unwounded cancerous swelling prevents it from future wounds.

Another topical anti-cancer drug is prepared as follows: pulverize red Armenian bole and sealing clay with the mixture of water and vinegar (or yoghurt) using lead mortar and pestle until the mixture becomes black. The resulting balm should be rubbed on the cancerous tumor. It is better to pulverize common houseleek and rose oil along with the above components (9). In some parts of his book, Ahwazi has discussed eye (Vol. 1, P. 340) and uterine (Vol. 1, PP. 86-87) cancers.

### 2.4. Avicenna

Avicenna was not only a physician but a great dignity in philosophy as well. The witness for this claim lies in his books: al-Shifa (The Recovery), al-Esharat wa al-Tanbihat (Remarks and admonitions), al-Naajat (Book of Salvation), 'Uyun al-Hikmah (Principles of Wisdom) and Daneshnameh-e-Alaii (Alaii’s Encyclopaedia). Avicenna wrote about 61 books and treaties in medical science including al-Adawiyah al-Qalbiyah (Cardiac Drugs), al-Orjozah fi al-Tibb (A Poethical Book in Medicine), al-Tashrih (Anatomy), al-Vasayah (Testament), and Resaleh Judiyah. Avicenna’s masterpiece is the book of “al-Qanun fi al-Tibb” (The Canon of Medicine) which is the mother book of medicine in the eastern and western worlds (1-6). Canon comprises 5 major books each divided into some arts, tuitions, sentences and chapters. 

The first book of Canon discusses the concept of medicine, particularly the medicine range and its subjects and also topics around humors, tempers, elements, organs, spirits, functions, and powers. Themes on diseases and their etiology, hygiene, and finally general guides to treatment are also mentioned. 

The second book is assigned to simple drugs and includes discussions about 800 mineral, herbal, and animal based medicinal materials. The drugs are ordered alphabetically (Abjad), and in each drug monograph, the manner, characteristics, the preferred drugs, nature, application, properties and indication are mentioned. The third book of Canon elaborates diseases cap-a-pie in 22 arts. Each art comprises several articles. In fact, this part acts as a complete review of pathology. The fourth book offers ways to cure general diseases such as fevers and edema, and also includes orthopedics, toxicology, and cosmetic and hygienic products 

The fifth and final book which is allocated to compound drugs is called Qarabadin and represents properties and recipes to make all kinds of pills, mixtures, powders, syrups, suppositories, tablets, and so on. There have been numerous expositions of whole Canon or its parts and it has been summarized many times. The book has been translated into European, Hebrew and Persian languages and it has been reprinted frequently. Avicenna has assigned a chapter of Canon to cancer (10). From his point of view, cancer is a kind of black bile swelling, which is caused by the black bile resulting from burning of the yellow bile. After mentioning differential characteristics of cancer and scirrhus, he adds that cancer frequently affects hollow organs and for this reason, its prevalence is higher among females. Highly innervated organs are more prone to cancer. At early stages, cancer growth is covert and latent. After progression, treatment of cancer would be difficult. Initially, cancer is the size of a broad bean or smaller, hard, spherical, dark and slightly warm. Some types of cancer are accompanied by severe pain, while others have mild pain, with some being mostly painless. Some cancers are prone to ulceration but in some other cases, ulcerative cancer could become non-ulcerative. In some occasions, manipulation of a tumor may lead to its ulceration. Naming of this disease as cancer (crab) might be due to the similarity between the shape of a cancerous tumor affecting an organ and a crab with its prey. The appellation may be also due to the sphericity and darkness of tumor and origination of vessels from its surroundings which resembles crab’s feet.

An important point in the treatment of cancer is that the progression and ulceration of cancerous tumor should be prevented as seriously as possible. Although some types of cancer could be treated during early stages of development, there is no possibility for the treatment of advanced cancers. In most occasions, cancer grows in viscera in a latent manner. In these cases, as Hippocrates mentiones, its irritation should be strictly avoided as it might lead to death. In contrast, when left untreated, the patient would have a longer life, especially if appropriate foods such as beer, soft-boiled egg yolk, and small river fishes are consumed. 

When the cancerous tumor is small, its removal is possible. If so, the tumor should be eradicated and some parts of adjacent normal tissues should also be excised in order to cut all tumor-feeding vessels. After cutting the cancerous tumor, bleeding should be allowed until large blood volumes come out of the body. Of course, purgation and venesection should have already been performed to cleanse the body from carcinogenic substances. Body cleansing should be performed using proper foods (from both qualitative and quantitative aspects) in order to prepare the organ for cutting. In some occasions, it may be necessary to cauterize the scar. However, if the cancerous tumor has come close to sensitive and vital organs, cauterization may be very dangerous. It has been quoted that a physician excised the cancerous breast of a woman but after a short time, her other breast became cancerous. For the purpose of purgation, either the mixture of dodder (18.75 g) and cheese whey or honey syrup, or decoction of dodder in oxymel should be administered once every few days. If the patient has good stamina, potion of black hellebore could be administered. Administration of topical anti-cancer drugs is performed to achieve the following 4 goals: 1. To achieve full cancer eradication, which is a very difficult task. 2. Preventing growth and progression of cancer; 3. Preventing ulceration of the cancerous tumor; and 4. Treatment of cancerous wounds.

Drugs that are used for the eradication of cancer should be able to dissolve the carcinogen and help excrete the dissolved carcinogen present in the cancerous organ. These drugs should not be strong or irritant because strong drugs increase cancer’s malignancy. Another requisite for these drugs is lack of caustic and irritant properties and taste. All these considered, it appears that washed mineral drugs are the best option. An example is washed zinc oxide mixed with oils such as Cheiranthus cheiri oil. In order to prevent growth and progression of cancer, the drug should reach the tumor’s body. On the other hand, modification of food and organ strengthening should be performed to prevent cancer progression. For this latter purpose, topical application of the following drugs is very beneficial: powdered grinding stone or knife grinder stone, liquid obtained from pulverization of lead in rose oil or coriander juice, and salving with unripe grape powder. Drugs that are administered for the prevention of tumor ulceration are also effective in halting tumor progression provided that they do not cause irritation. These drugs are especially effective if administered in combination with the liquid obtained from lead pulverization. The aforementioned drugs include sealing clay, red Armenian bole, unripe olive oil, common houseleek juice, basic carbonate of lead with L. sativa extract and psyllium (Plantago psyllium L.) mucilage. Another drug for this purpose, which is among the best ones, is the poultice prepared from mashed fresh caught river crab especially with litharge of gold or silver. Treatment methods that are employed for the healing of cancerous wounds include: 1. Dressing with a cotton cloth soaked in black nightshade juice. When dried, the cloth should be moistened with the mentioned water; 2. A mixture of wheat kernel (3.34 g), frankincense (3.34 g), basic carbonate of lead (3.34 g), sealing clay (6.68 g), red Armenian bole (6.68 g), and washed aloe solid extract (6.68 g) should be prepared and pulverized. If the cancerous wound is wet, the powder should be sprinkled but if the wound is dry, the powder should be mixed with rose oil and converted to balm before application. Topical application of the mixture prepared from crab ash and ghiroty (mixture of wax and rose oil) is also useful. Likewise, topical application of drug prepared from washed zinc oxide and P. oleracea water (or P. psyllium) may prove beneficial.

### 2.5. Jorjani

Jorjani wrote valuable books in medicine during his lifetime. His biggest treasure is the detailed book of Zakhireh Kharazmshahi (Treasure of Kharazmshah). Zakhireh is the most important medical book in Persian. The book contains nine main books and two appendices on simple and compound drugs.

The first book of Zakhireh is about medical science, identifying types of humors and temperaments, and also general aspects of anatomy.

The second book discusses health and diseases and also types of pulses, sweating, urine and feces.

The third book includes a complete course on maintaining health.

The forth book is allocated to ways of diagnosing disease and disease duration.

The fifth book is on identifying different types of fevers and methods to cure them.

The sixth book is assigned to methods of curing diseases cap a pie. 

The seventh book describes types of inflammation, wounds, and fractures, and their treatments.

The eighth book includes cosmetics and beautification.

The ninth book is assigned to types of poisons, antidotes, bites, venerations, and their treatments.

The final section explains simple and compound drugs in detail.

In fact, this very book can be regarded as an encyclopaedia fraught with pure Persian medico- pharmaceutical terms. Zakhireh is also noticeable in literary. 

The complete text of Zakhireh was photo-printed in 1976 by the Iranian Culture Foundation. Some of the book volumes were published incompletely. Due to its importance, Zakhireh was translated into Hebrew and Turkish. 

Jorjani wrote a summary of the Zakhireh named al-Aqraz al-Tibbiah wa al-Mabaheth al-Alaiah (Medical Goals and Alaiyeh’s Discussions). This collection contains five books. The first offers an introduction to medicine, the second book is about public health, the third one discusses disease treatment cap-a-pie, the forth book comprises simple drugs and finally the fifth book is assigned to evaluation of compound drugs. The photo-print of al-Aqraz al- Tibbiah was published in 1966 by the Iranian Culture Foundation. Fortunately, this book was edited by Professor H. Tadjbakhsh and published by Tehran University press in 2006. The third book of Jorjani in medicine is named “Khofi Alaii” (Hidden book of Alaii), which is an abbreviated medical text and has two parts. The first part includes the theoretical aspects of medicine and is of two articles. The second is a scientific medical knowledge and comprises seven articles. The book was lithographed in Kanpur in India in 1891. It was also published with valuable footnotes and descriptions by Etela’at Institute located in Tehran. 

The forth book of Jorjani namely “Yadegar” (The Keepsake) is an extract text and codified in five parts. The first part has 17 chapters, the second includes 30 chapters, the third contains 2 chapters, the forth is comprised of 11 chapters and finally the fifth is inclusive of 3 chapters. Yadegar was edited by Professor M. Mohaghegh and published by the Institute of Islamic Studies in Tehran in 2003. One of Jorjani’s essays is “Zobdat al-Tibb” (Selected Topics in Medicine). The book was written in Arabic, and its context was ordered in numerous tables. This book is yet to be printed (2, 3, 5, 6).

Jorjani has assigned parts of Zakhireh to cancer (11). In his point of view, cancer is a kind of black bile swelling which is, unlike scirrhus, accompanied by pain, pulsation, inflammation and angiogenesis. These characteristics could be applied for the differential diagnosis of cancer from scirrhus. In addition, vessels of cancerous tissue have a dark green color. Cancer is frequently formed in soft and porous organs and for this reason, it mainly affects breast and innervated organs (such as uterus) in females, and throat, larynx, testicles and penis in males. Intestine is another organ which is prone to cancer. Diagnosis of cancer in the early stages is difficult. On the other hand, upon progression and appearance of clinical manifestations, treatment of cancer would be difficult. Cancerous tumor is initially hard, dark colored, slightly warm and in the size of a broad bean or smaller. In some cases, cancer is accompanied by severe or mild pain. Some cancerous tumors are easily ulcerated whereas some other is not. In some occasions, application of appropriate drugs would prevent the ulceration of susceptible tumors. In contrast, administration of some substances may ulcerate cancerous tumors that are not considered to be prone to ulceration.

Jorjani has mentioned that stabilization and prevention of cancer progression should be attempted. In addition, ulceration of cancerous tumor should also be avoided. If treatment is started during early stages of cancer development, recovery is possible but advanced cancers are not treatable. In most cases, development and progression of cancer in visceral organs is a gradual process. For such cases, manipulation and treatment of tumor should be avoided as these may cause irritation and progression of the disease and eventually lead to the shortening of the patient's life. Conversely, lack of manipulation, using appropriate diets and timely evacuation would increase the longevity of patients. For these patients, foods like beer, almond oil, soft-boiled egg yolk, vetch (Phaseolus mungo L.), spinach (Spinacia oleracea L.), and pumpkin are administered. In cases with high temperature, administration of fresh cow's dough (prepared after isolation of butter) is beneficial. Such dough should be consumed before becoming sour. In some occasions, small cancerous tumors which are distant from vital organs may be removed via surgery. If so, the tumor should be cut from its origin together with some parts of adjacent normal tissues. In addition, bleeding should be allowed until large blood volumes come out. Afterwards, the injured site should be salved. In some cases, the organ is cauterized after cutting the tumor. It must be noted that cutting the tumor may be perilous in the majority of cases.

To treat the aforementioned complication, the body must first be cleansed from black bile. For this purpose, 13.36 g of dodder in cheese whey or honey syrup is administered once every few days. Among mineral drugs, washed zinc oxide is beneficial especially if rubbed with rose oil on the tumor. Likewise, rubbing the liquid obtained from pulverization of lead pieces in chicory juice, coriander juice or lettuce extract on tumor could prevent its enlargement and ulceration. Other useful medications include poultices of basic lead carbonate, aloe, red Armenian bole, sealing clay, common houseleek extract and ispaghula mucilage. In another part (PP. 562-563) of his book, Jorjani has explained the signs and treatment methods of uterine cancer as well as effective drugs against this type of cancer.

## 3. Conclusions

The common point of all assessed books in the present review is the pivotal role of black bile in the development of cancer. Therefore, all listed physicians have insisted on the prominent impact of black bile purgatives in cancer therapy. All evaluated medical books implied that in case of advanced cancers with progression (metastasis) to other tissues, organectomy is the only therapeutic measure, followed by eradication of all cancer roots and adjacent vessels. Finally, all physicians mentioned in the present review consistently relied on the use of herbal medicine for the treatment of cancer or halting its progression (Table 1). It is greatly recommended that further research be undertaken to explore the contents of modern scientific literature on the anti-cancer properties of medicinal plants mentioned in the major books of Islamic traditional medicine.

**Table 1:** List of Medicinal Plants Mentioned in the Current Review for the Treatment of Cancer and its Complications

Scientific name	Common name	Application
*Erysimum officinale* L.	Hedge mustard	Non-ulcerative cancer
*Lepidium sativum* L.	Garden cress	Indurated swellings of breasts and testicles
*Urtica dioica* L.	Nettle	Corrosive cancers
*Juglans regia* L.	Walnut	Ulcerative melatonic swellings
*Cichorium intybus* L.	Chicory	ulcerative, pulsating and warm cancers; cleansing the body from black bile
*Anethum gravolens* L.	Dill	Newly developed cancers
*Cicer arientium* L.	Pea	Healing of cancerous wounds
*Salix caprea* L.	Goat willow	Healing of cancerous wounds
*Platanus orientalis* L.	Oriental plane tree	Healing of cancerous wounds
*Malva rotundifolia* L.	Dwarf mallow	Healing of cancerous wounds
*Cuscuta epithymum* Murr.	Clover dodder	Boosting organ's function and black bile removal
*Chenopodium album* L.	Squarters goosefoot	Cancer treatment or halting its progression
*Cucurbita pepo* L.	Pumpkin	Cancer treatment or halting its progression; black bile purgative; increasing the longevity of cancer patients
*Boswellia carteri* Birdew.	Frankincense	Healing of cancerous wounds
*Aloe* spp.	Aloe	Healing of cancerous wounds
*Solanum nigrum* L.	Black nightshade	Healing of cancerous wounds; cleansing the body from black bile; healing of tumor injury
*Latuca sativa* L.	Lettuce	Halting tumor progression; healing of cancerous wounds
*Sempervivum tectorum* L.	Common houseleek	Halting tumor progression; healing of cancerous wounds
*Rhus coriaria* L.	Sumac	Treatment of corrosive cancers; healing of cancerous wounds
*Portulaca oleracea* L.	Purslane	Treatment of corrosive cancers; healing of cancerous wounds
*Cinnamomum cassia* (L.) J.Presl	Cassia	Healing of corrosive cancerous wounds
*Cupressus sempervirens* L.	Mediterranean cypress	Healing of corrosive cancerous wounds
*Matthiola incana* (L.) W.T.Aiton	Hoary stock	non-ulcerative cancers
*Arum dracunculus* L.	Dragon wort	treatment of chronic cancers
*Eruca sativa* Mill.	Rocket	non-ulcerative cancers
*Gundelia tournefortii* L.	Gundelia	Treatment of cancerous swellings
*Linum usitatissimum* L.	Flax	Treatment of cancerous swellings
*Brassica oleracea* L.	Cabbage	treatment of cancer
*Plantago major* L.	Common plantain	treatment of cancer
*Plantago ispaghula* Roxb.	Ispaghula	Prevention of the progression and injury of the tumor
*Althaea officinalis* L.	Marsh-mallow	Healing of tumor injury
*Cinnamomum camphora* (L.) T.Nees & C.H.Eberm.	Camphor	Healing of tumor injury
*Polyporus officinalis* Fries	White agaric	Black bile purgative
*Terminalia chebula* Retz.	Black myrobalan	Cleansing the body from black bile
*Polypodium vulgare* L.	Common polypody	Cleansing the body from black bile
*Lavandula stoechas* L.	French lavender	Cleansing the body from black bile
*Helleborus niger* L.	Black hellebore	Cleansing the body from black bile
*Amaranthus blitum* L.	Blite	Black bile purgative; healing of tumor injury
*Physalis alkekengi* L.	Bladder cherry	Cleansing the body from black bile
*Coriandrum sativum* L.	Coriander	Healing of tumor injury
*Plantago psyllium* L.	Psyllium	Halting tumor progression; healing of cancerous wounds
*Phaseolus mungo* L.	Vetch	Increasing the longevity of cancer patients
*Spinacia oleracea* L.	Spinach	Increasing the longevity of cancer patients
